# WGX50 mitigates doxorubicin-induced cardiotoxicity through inhibition of mitochondrial ROS and ferroptosis

**DOI:** 10.1186/s12967-023-04715-1

**Published:** 2023-11-17

**Authors:** Panpan Tai, Xinyu Chen, Guihua Jia, Guanjun Chen, Lian Gong, Yaxin Cheng, Zhuan Li, Heng Wang, Aiyan Chen, Ganghua Zhang, Yuxing Zhu, Mengqing Xiao, Zhanwang Wang, Yunqing Liu, Dongyong Shan, Dong He, Moying Li, Tianzuo Zhan, Abbas Khan, Xiaohui Li, Xiangxiang Zeng, Chaopeng Li, Dongsheng Ouyang, Kelong Ai, Xuan Chen, Dongbo Liu, Zhonghua Liu, Dongqing Wei, Ke Cao

**Affiliations:** 1 Department of Oncology, Third Xiangya Hospital, Central South University, Changsha, China; 2 School of Life Sciences and Biotechnology, Shanghai Jiao Tong University, Shanghai, China; 3 The Key Laboratory of Model Animals and Stem Cell Biology in Hunan Province, Hunan Normal University School of Medicine, Changsha, 410013 China; 4 The Key Laboratory of Study and Discovery of Small Targeted Molecules of Hunan Province, Hunan Normal University School of Medicine, Changsha, 410013 China; 5 Department of Pharmacy, Hunan Normal University School of Medicine, Changsha, 410013 China; 6 Staff Hospital of Central South University, Central South University, Changsha, China; 7 Department of Medicine II, Medical Faculty Mannheim, Heidelberg University, Mannheim, Germany; 8 Department of Pharmacology, Xiangya School of Pharmaceutical Sciences, Central South University, Changsha, China; 9 College of Computer Science and Electronic Engineering, Hunan University, Changsha, China; 10 Hunan Key Laboratory for Bioanalysis of Complex Matrix Samples, Changsha Duxact Biotech Co., Ltd, Changsha, China; 11 Department of Clinical Pharmacology, Xiangya Hospital, Central South University, Changsha, China; 12 Xiangya School of Pharmaceutical Sciences, Central South University, Changsha, China; 13 Hunan Provincial Key Laboratory of Cardiovascular Research, Xiangya School of Pharmaceutical Sciences, Central South University, Changsha, China; 14 College of Horticulture, Hunan Agricultural University, Changsha, China; 15 State Key Laboratory of Subhealth Intervention Technology, Changsha, China; 16 National Research Center of Engineering Technology for Utilization Ingredients From Botanicals, Changsha, China

**Keywords:** WGX50, DOX-induced cardiotoxicity, Mitochondrial ROS, GPX4, Ferroptosis

## Abstract

**Background:**

Doxorubicin (DOX)-induced cardiotoxicity (DIC) is a major impediment to its clinical application. It is indispensable to explore alternative treatment molecules or drugs for mitigating DIC. WGX50, an organic extract derived from *Zanthoxylum bungeanum* Maxim, has anti-inflammatory and antioxidant biological activity, however, its function and mechanism in DIC remain unclear.

**Methods:**

We established DOX-induced cardiotoxicity models both in vitro and in vivo. Echocardiography and histological analyses were used to determine the severity of cardiac injury in mice. The myocardial damage markers cTnT, CK-MB, ANP, BNP, and ferroptosis associated indicators Fe^2+^, MDA, and GPX4 were measured using ELISA, RT-qPCR, and western blot assays. The morphology of mitochondria was investigated with a transmission electron microscope. The levels of mitochondrial membrane potential, mitochondrial ROS, and lipid ROS were detected using JC-1, MitoSOX™, and C11-BODIPY 581/591 probes.

**Results:**

Our findings demonstrate that WGX50 protects DOX-induced cardiotoxicity via restraining mitochondrial ROS and ferroptosis. In vivo, WGX50 effectively relieves doxorubicin-induced cardiac dysfunction, cardiac injury, fibrosis, mitochondrial damage, and redox imbalance. In vitro, WGX50 preserves mitochondrial function by reducing the level of mitochondrial membrane potential and increasing mitochondrial ATP production. Furthermore, WGX50 reduces iron accumulation and mitochondrial ROS, increases GPX4 expression, and regulates lipid metabolism to inhibit DOX-induced ferroptosis.

**Conclusion:**

Taken together, WGX50 protects DOX-induced cardiotoxicity via mitochondrial ROS and the ferroptosis pathway, which provides novel insights for WGX50 as a promising drug candidate for cardioprotection.

**Graphic abstract:**

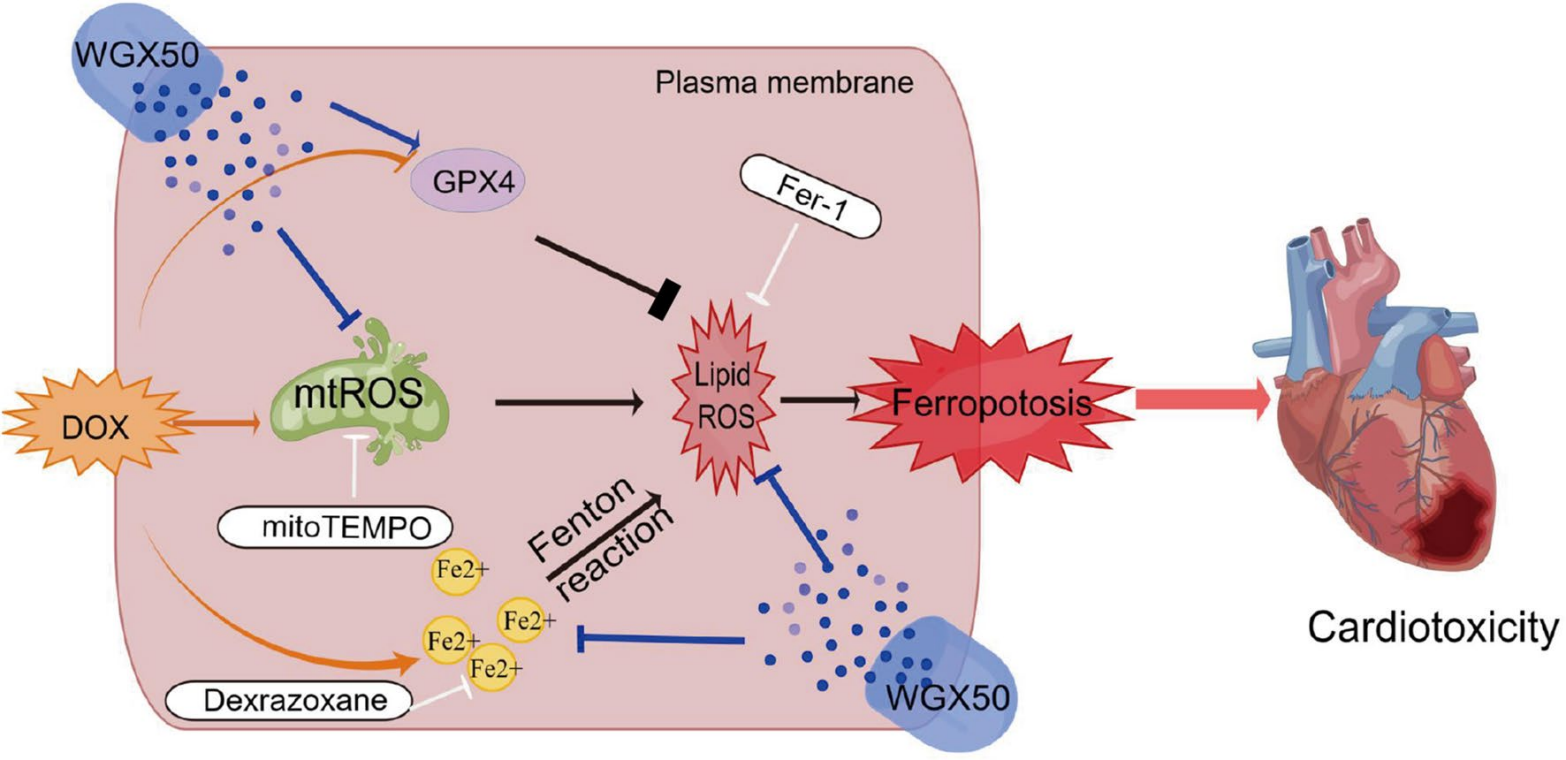

**Supplementary Information:**

The online version contains supplementary material available at 10.1186/s12967-023-04715-1.

## Introduction

Doxorubicin (DOX), a class of anthracycline anticancer drugs, is widely used to treat a variety of solid and hematologic malignancies. However, its clinical application has been severely limited due to DOX-induced cardiotoxicity (DIC) [1, 2]. DIC is dose-dependent, and clinical studies show that 26% of patients will experience drug-related congestive heart failure (CHF) once a cumulative DOX dose of 550 mg/m^2^ is reached [3]. DOX-induced irreversible myocardial damages, such as degenerative cardiomyopathy and CHF, endangers human life [4].

The definite mechanism of DIC remains unclear but it involves in different biological processes, such as reactive oxygen species (ROS) production, mitochondrial iron accumulation, topoisomerase inhibition, disruption of mitochondrial biogenesis, degradation of myofibrils, and multiple forms of cell death [5–8]. Among these, the generation of ROS is considered the most important. After anthracycline treatment, there is a considerable rise in ROS in cardiomyocytes, resulting in severe myocyte damage [9, 10]. This is highly relevant to the quinone structure of DOX, which can be reduced to semi-quinone radicals in cardiomyocytes. Increased oxygen consumption readily facilitates semi-quinone transformation generating large amounts of ROS [11]. Since 2012 when Dixon initially proposed the concept of ferroptosis, there has been an increasing number of studies on ferroptosis in various diseases. At present, Kai Hou and Yilong Wang et al. have pointed out the association of ferroptosis in DIC [12, 13]. As an iron-dependent cell death, ferroptosis is featured by lipid peroxidation as one facet. Avid phospholipid peroxidation within the plasma membrane causes damage to the cardiomyocyte and consequently results in severe cardiac impairment and dysfunction. Excess ROS and imbalanced iron metabolism are the major driving forces for this process [14]. In addition, DOX does generate ROS and disrupt mitochondrial function by interacting with intracellular iron balance [15].

Other than ferroptosis in DIC, mitochondria are the major organelle that is involved in the process of phospholipid peroxidation [16]. Anthracyclines primarily accumulate in mitochondria after entering the body [17]. Cardiolipin is abundant in the inner mitochondrial membrane, for which DOX has a specific higher affinity. The irreversible binding disrupts the integrity of the mitochondrial membrane and compromises the functionality of the organelle [6]. Anthracycline increases iron levels that preferentially accumulate in specific subcellular compartments, particularly in cardiac mitochondria, leading to cardiotoxicity [18]. Besides, anthracyclines can be reduced to the semi-quinone form in mitochondria generating large amounts of ROS [18]. In cardiac tissue, mitochondria are more abundant as powerful energy supplier, which are not only an essential source of ROS but also a target for ROS, leading to particularly high vulnerability of the heart to DOX [19].

As an iron chelator, Dexrazoxane remains the only ameliorant that has been approved by the U.S. Food and Drug Administration (FDA) to reduce incidences of DIC in chemotherapy recipients [20]. However, it still has unexpected events owing to its counteraction to the antitumor activity of DOX and the possible induction of secondary malignant lesions. Various organic extracts have been proven in recent research to be beneficial in the treatment of DIC, such as Daidzein [21], Salidrosid [22], Psoralidin [23], *Amauroderma* *rugosum* [24], and other plant drugs. However, the clinical therapeutic potential of these organics requires further investigation. Therefore, there is an urgent need to keep exploring the mechanisms of DIC and to discover safer and more effective compounds to treat DIC. *Zanthoxylum bungeanum* Maxim is a food flavoring and traditional herbal medicine in China and is documented in Chinese Pharmacopoeia (ChP, 2020). Its long-standing use has well-endorsed its efficacy in analgesic, anti-inflammatory, antioxidant, and antibacterial activities [25–27]. WGX50, a small molecule entity derived from *Zanthoxylum bungeanum* Maxim, has similar anti-inflammatory and antioxidant effects as *Zanthoxylum bungeanum* Maxim in previous studies [28–30]. Moreover, WGX50 also inhibits neuroinflammation induced by the amyloid-beta (Aβ) peptide in microglia via 7nAChR activation of the JAK2/STAT 3 and PI3K/AKT pathways, which may be a hopeful therapeutic molecular compound against Alzheimer’s Disease (AD) [31–34]. Furthermore, as a novel drug candidate for AD, WGX50 directly reduces accumulation of Aβ oligomers in cerebral cortex [34] and protects neurons from damage triggered by Aβ stimulus via TLR4-mediated NF-κB and MAPK signaling pathways in primary microglia and transgenic mice [35]. However, the effects of WGX50 on DOX-induced myocardial injury have not been investigated. Herein we examined the potential protective effects of WGX50 in DIC models, hypothesizing that it ameliorates DIC by its anti-ferroptosis activity.

Our study provides novel insights into cardioprotective effects of WGX50 against DOX-induced cardiotoxicity with solid evidence in both DOX-induced myocardial injury mouse models and cardiomyocytes. Our findings reveal that WGX50 regulates ferroptosis and alleviates DIC via inhibiting iron accumulation, mitochondrial ROS, and lipid peroxidation in cardiomyocytes and mice. We therefore conclude that WGX50 supplement could help to reduce cardiotoxicity while maximizing the antitumor effect of DOX as a valuable option for clinical treatment of DIC.

## Materials and methods

### DOX-induced mouse DIC model

Male C57BL/6 mice (age: 8–10 weeks, weight: 20–25 g) were purchased from Hunan SJA Laboratory Animal Co., Ltd (Changsha, Hunan). All mice were kept in specific pathogen-free facilities on a 12-h day and night cycle at 23 °C. After being habituated the environment for 1 week, all mice were randomly divided into 5 groups (n = 5): Control group (Control), DOX treatment group (DOX), DOX treatment plus Fer-1 treatment group (DOX + Fer-1), DOX treatment plus WGX50 treatment group (DOX + WGX50) and DOX treatment plus mitoTEMPO treatment group (DOX + mitoTEMPO). WGX50 was provided by Professor Dongqing Wei’s laboratory at Shanghai Jiao Tong University. Mice in DOX group and control group were given DOX (ip.5 mg/kg, MedChem Express, USA) or an equal volume of saline plus solvent mixture once a week for 4 weeks [36]. Mice in DOX + WGX50 group were treated with WGX50 (ip.1 mg/kg/d) for 4 weeks plus DOX administration [35]. Mice in DOX + mitoTEMPO group were treated with mitoTEMPO (ip.0.7 mg/kg/d, Adooq Bioscience, USA) for 4 weeks plus DOX administration [37]. Mice in DOX + Fer-1 group were given Fer-1 (i.p.1 mg/kg, Adooq Bioscience, USA) once a day after DOX administration for a total of 4 weeks [38]. All reagents were dissolved in DMSO and diluted to the appropriate concentration with normal saline. Mice were then killed after transthoracic echocardiography examination. Tissue or organs were then collected, fixed with 4% paraformaldehyde or frozen in liquid nitrogen followed by storage at − 80 °C for further experiments. All applicable international, national, and/or institutional guidelines for the care and use of animals were followed.

### Cell culture and drug treatment

Mouse cardiomyocyte HL-1 and rat cardiomyocyte H9C2 were purchased from Shenzhen Haodi Huatuo Biotechnology Co., LTD (Shenzhen, China). The cells were cultured in Dulbecco's modified Eagle medium (DMEM, Gibco, Australia) supplemented with 10% fetal bovine serum (FBS, Gibco, Australia) and 1% penicillin–streptomycin (Gibco, Australia) at 37 °C in a humidified incubator with 5% CO2. HL-1 and H9C2 cells were exposed to DOX (HL-1:2 μM, H9C2:1 μM) while subjected or not to 10 μM Fer-1(Adoop, USA), 5 μM mitoTEMPO (Adoop, USA), 5 μM NAC (Adoop, USA) and WGX50 (Provided by Professor Dongqing Wei’s Laboratory of Shanghai Jiao Tong University) with suitable concentration co-treatment for appropriate time. Erastin (HL-1:5 μM, Adoop, USA) was used to verify the effect of WGX50 on ferroptosis. Then proceed to the next experiment.

### Echocardiography

After isoflurane anesthetizing and immobilizing mice, we measured M-curves along the short axis of the papillary muscle and at the level of the left ventricular section using the Vevo 2100 microultrasound imaging system (VisualSonics, Canada). Based on these measurements, we calculated the left ventricular ejection fraction (LVEF, EF%) and left ventricular fractional shortening (LVFS, FS%) of mice. EF (%) = (LVDV − LVSV)/LVDV × 100%; FS (%) = (LVDD − LVSD)/LVDD × 100%. LVDV: Left Ventricular Diastolic Volume, LVSV: Left Ventricular Stroke Volume, LVDD: Left Ventricular end Diastolic Diameter, LVSD: Left Ventricular end Systolic Diameter. Three measurements were taken and the mean value was calculated.

### Histology

First, mouse myocardial tissue was fixed in 10% formalin at 4 °C overnight. Then the samples were dehydrated, infiltrated, embedded in paraffin, and cut into 5 μm-thick tissue sections by pathological microtome. Finally, Hematoxylin and Eosin (HE) staining and Masson staining were performed on the tissue sections.

### Transmission electron microscope

Fresh myocardial samples with the size of 2 mm3 were collected and immediately fixed in electron microscope solution for 4 h. Then the samples were fixed with 1% osmic acid in·0.1 M phosphate buffer solution (PBS, pH 7.4) at room temperature (20 °C) for 2 h. Rinse with PBS (pH 7.4) 15 min each time and repeat for 3 times. 50%–70%–80%–90%–95%–100%–100% alcohol–100% acetone–100% acetone ascending dehydration, 15 min each time. After the samples were permeated with acetone, they were encapsulated by 60 °C oven polymerizations for 48 h. Use an ultra-thin microtome (Leica, Switzerland) to slice 60–80 nm ultra-thin sections. The slices were stained with uranium lead (2% uranium acetate saturated alcohol solution and lead citrate for 15 min each) and dried overnight at room temperature. Observation was made under a transmission electron microscope (Hitachi, Japan), and the images were collected and analyzed.

### Cell viability

The viability of HL-1 cells after treatment was determined by CCK-8 kit (Beyotime Biotechnology, Shanghai, China). In short, after HL-1 cells from different treatment groups were incubated for 24 h, cells were washed 3 times with PBS solution at pH 7.4. After that, a mixture of 90 μL of medium and 10 μL of CCK-8 reagent was added into each well (96-well plate) and cells were cultured accordingly at 37 °C for an additional 3.5 h. The optical density at 450 nm wavelength was measured. Cell relative survival rate (%) was calculated.

### Measurement of Fe^2+^ and MDA

The content of Fe^2+^ in mouse heart tissue and HL-1 and H9C2 cells were determined by an iron assay kit (Nanjing Jiancheng Bioengineering Institute, Nanjing, China). Cardiac tissue homogenates or cell lysates were centrifuged at 12, 000 rpm for 10 min at 4 °C, then the supernatant was collected and an aliquot of them was thoroughly mixed with a set of reagents as manufacturer's instruction, and thereafter letting them stand at room temperature for 15 min. Afterwards, 200 μL of supernatant was taken into a 96-well plate, and the absorbance was recorded at 562 nm using a microplate reader. Malondialdehyde (MDA) level in mice heart tissue was quantitated using MDA kits (Beyotime, Shanghai, China) according to the manufacturer’s instructions.

### Measurement of ATP and GSH

10 mg of myocardial tissue samples or about 1 × 106 cell samples were lysed with 100 μL ATP Buffer, the resulting lysates were centrifuged at 15,000 rpm for 2 min to collect supernatant. ATP content was determined using an ATP Assay kit (Biovision, USA) according to manufacturer's instructions. The GSH and GSSG detection kit (Beyotime, Shanghai, China) was used to measure the content of GSH and GSSG as manufacturer's instructions, and the ratios of GSH/GSSG were calculated.

### Myocardial damage assay and RT-qPCR

Mouse myocardial tissue homogenates and HL-1/ H9C2 cell lysates were assayed for cTnT and CK-MB using corresponding assay kits (Nanjing Jiancheng Bioengineering Institute, Nanjing, China) as manufacturer's instructions.The levels of ANP and BNP in mouse myocardium and HL-1cells were detected using reverse transcription quantitative polymerase chain reaction (RT-qPCR). Total RNA was extracted from mouse heart tissues and cells using TRIzol reagent (Invitrogen, USA). 1 µg total RNA was reverse-transcribed into cDNA using a reverse transcription kit (Vazyme, Nanjing, China), and qPCR was performed using SYBR GreenPCR Master Mix (Vazyme, Nanjing, China) to determine the mRNA expression levels of ANP and BNP. 2-ΔΔCT method was used to calculate the relative gene expression and β-actin was used as internal control. Primers are as follows: ANP, Forward: 5′-GTGCGGTGTCCAACACAGAT-3′; Reverse, 5′-TCCAATCCTGTCAATCCTACCC-3′. BNP, Forward: 5′-AGTCCTTCGGTCTCAAGGCA-3′; Reverse, 5′-CCGATCCGGTCTATCTTGTGC-3′. β-actin, Forward, 5′-GTGACGTTGACATCCGTAAAGA-3′; Reverse, 5′- GCCGGACTCATCGTACTCC-3′.

### JC-1 fluorescence staining

Mitochondrial membrane potential (MMP) was detected by JC-1 staining assay kit (Beyotime, Shanghai, China). According to the manufacturer's instructions, HL-1 or H9C2 cells were incubated with JC-1 reagent for 20 min. After washing HL-1 and H9C2 cells with JC-1 staining buffer, the cells were observed and images were collected through the fluorescence microscope (Zeiss, Germany). Once the MMP is higher, JC-1 accumulates in the mitochondria matrix forming polymer which produces red fluorescence (λex = 525 nm; λem = 590 nm); On the contrary, JC-1 will accumulate in the matrix of mitochondria as monomer generating green fluorescence (λex = 490 nm; λem = 530 nm).

### Detection of mitochondrial ROS

MitoSOX™ (Invitrogen, USA) red dye is living-cell permeant and is capable of selectively targeting mitochondria where once it was oxidized by superoxide, it would produce red fluorescence (λex = 400 nm; λem = 590 nm). HL-1 and H9C2 cells were cultured in 6-well plates, after reaching 70–80% confluency, the medium was discarded, and the cells were washed with PBS for 3 times. Add 1 mL of 5 μM MitoSOX solution and incubate at 37 °C for 10 min. Then, cells were washed again with PBS for 3 times, and dyed with 1 μg/mL Hoechst for 10 min. Confocal fluorescence microscope (Zeiss, Germany) was used for observation and photo analysis.

### Detection of lipid peroxidation

Lipid peroxidation levels were tested using the C11-BODIPY 581/591 kit (Invitrogen, USA). HL-1 and H9C2 cells were inoculated into 6-well plates, and cultured to proliferate to about 5 × 104 cells per well. The medicated cells were then incubated with 5 μM BODIPY (λex = 581 nm, λem = 591 nm) probe at 37 °C for 30 min according to manufacturer's instructions. Fluorescence was measured using a fluorescence microscope (Zeiss, Germany), which also was utilized for visualization. Fluorescence imaging was performed using conventional filters, they’re Texas Red (581/591 nm) and FITC (488/510 nm). In the meanwhile, data acquisition was performed readily for oxidized BODIPY at the excitation and emission maxima of 488 nm and 510 nm, respectively.

### Western blotting

In brief, total protein was extracted from mouse myocardium, HL-1 and H9C2 cells using a protein extraction kit (Beyotime, Shanghai, China), and the concentration of which was quantified using a BCA assay kit (Beyotime, Shanghai, China). Proteins in each sample were separated by SDS-PAGE (Sigma, Germany) and were transferred to a polyvinylidene fluoride membrane (Millipore, Germany). The membrane was then blocked with 5% skim milk powder (Sangon Biotech, Shanghai, China), and incubated with primary antibody overnight at 4 °C: GPX4 (1:500; Proteintech, Wuhan, China), TOM20 (1:2000; Proteintech, Wuhan, China). Subsequently, the membrane was subjected to the incubation with goat anti-rabbit IgG-horseradish peroxidase conjugated secondary antibody (1:4000; Proteintech, Wuhan, China) after 3 times TBST washing. β-actin (1:2000; Proteintech, Wuhan, China) was used as the internal control. Finally, Immunoassay was performed by an enhanced chemiluminescence detection system (ECL; Biosharp, BL520A) combined with a Western blot system (Auragene). The expression of the target band relative to the loading control was quantified with integrated density by ImageJ software.

### Molecular docking and simulation of WGX-50 with GPX4

To understand the binding and interaction mode of WGX-50 with GPX4, we performed molecular docking of WGX-50 with the active site of GPX4 protein. For the docking purpose, AutoDock Vina software was used. The docking grid was generated based on W21, W23, V98, F100, and M102 residues as described by previous literature [39]. A docking grid parameters were based on the aforementioned residues and identified as: Centre (X = -24.0; Y = 9.30, and Z = 3.00) while the grid dimensions were set as: size (X = 16.00; Y = 16.00, Z = 16.00) respectively. Furthermore, default parameters were used for the docking. An unbiased fully atomistic simulation was conducted for the WGX-50-GPX4 complex. The simulations were performed using the PMEMD.CUDA code with the AMBER21 software [40–42]. To optimize the simulation protocol, the following steps were taken: first, gentle minimization was applied to the water molecules with 10,000 steps of steepest descent and 6000 steps of conjugate gradient minimization, while keeping the other atoms restrained. Then, the entire system was minimized without any constraints using the same parameters as before. The systems were gradually heated up to 300 K for 1000 ps, using a NPT approach to relax the positional restraints of the solvent molecules. The whole system was then relaxed for 1 ns using the NPT ensemble with a temperature of 300 K and no restraints. The Langevin thermostat was used with a collision frequency of 1.0/ ps, applying a pressure of 1 bar and a relaxation time of 4 ps. The systems were equilibrated for 50 ns using the NVT ensemble, treating the interactions between heavy and hydrogen atoms with the SHAKE algorithm. Electrostatic contacts were treated with a particle-mesh Ewald sum approach, using a cut-off of 10.0 Å for non-bonded interactions and Lennard–Jones interactions, while considering the real space part of the electrostatic interactions of the Ewald summation approach. Finally, a production run of 200 ns for each complex was completed using the NVT ensemble with a temperature of 300 K [40, 43]. The retained interactions, structural stability and perturbations, and functional variance caused by the most dynamic regions were assessed using the CPPTRAJ and PTRAJ modules of AMBER21[44]. To estimate the binding strength during molecular simulation, the MM/PBSA.py script was utilized, which allows the quantification of binding free energy. This method is widely used and has advantages over other methods, as it is computationally inexpensive and less time-consuming [45–48]. The script was applied to each complex, and the vdW, electrostatic, SASA, and G.B. components were calculated using the entire simulation trajectory.

### Statistical analysis

Data was analyzed using GraphPad Prism 9.0.0 (GraphPad Software Inc., San Diego, California, USA). The data conform to normal distribution and homogeneity of variance. All readouts were expressed as mean ± standard deviation (SD). Two-tailed unpaired Student t-test was used to evaluate statistical significance of difference between two intervention groups. In accordance, one-way ANOVA (analysis of variance) was performed among three or more groups. Significance level was set as p values < 0.05.

## Results

### WGX50 alleviates DOX-induced myocardial injury and ferroptosis in C57BL/6 mice

**Fig. 1:**
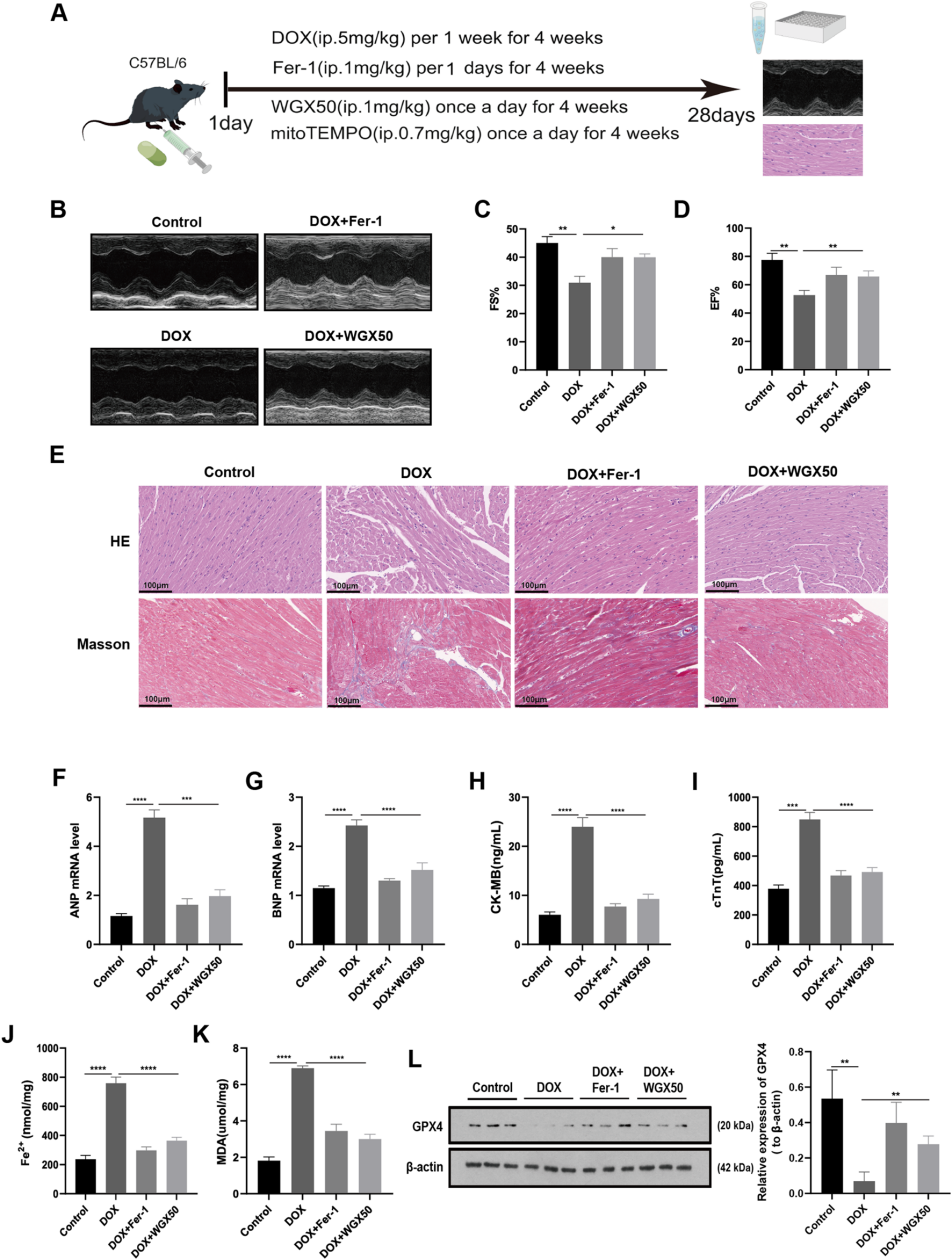
Doxorubicin-induced cytotoxicity and cardiomyocyte ferroptosis were relieved by WGX50 in C57BL/6 mice. **A** Schematic diagram of animal experiments. By Figdraw. **B** Echocardiographic photographs of M-mode in C57BL/6 mice. **C**, **D** Bar charts of left ventricular FS% and EF% in C57BL/6 mice. **E** Images of HE and Masson-stained mouse myocardium, “_” represents 100 µm. **F**, **G** The levels of ANP and BNP in myocardial tissues were detected by RT-qPCR. **H**–**K** ELISA kits were used to detect the levels of CK-MB, cTnT, Fe^2+^ and MDA in mice myocardial tissue. **L** Western blot bands represent GPX4 protein expression in mouse tissues of different groups, and the histogram stands for quantitative data of GPX4 normalized by β-actin. Values are presented as the mean ± SD of three individual experiments (n = 3). *, **, ***, and **** respectively means *p* < 0.05, *p* < 0.01, *p* < 0.001, and *p* < 0.0001

Additional file 1: Fig. S1A shows the structural formula of WGX50. The administration of WGX50 in animals was found to be safe as evidenced by the absence of significant toxicity in major organs in C57BL/6 mice following daily intraperitoneal injection of 1 mg/kg WGX50 for one month (Additional file 1: Fig. S1B). Ferroptosis is a form of cell death that plays a fundamental role in DIC [15]. Here we have investigated the effects of WGX50 on ferroptosis in DIC. To this end, we employed C57BL/6 male mice to construct a DIC model, as depicted in Fig. 1A. Echocardiography revealed significant reductions in left ventricular systolic fraction (FS%) and ejection fraction (EF%) in mice when subjected to DOX intervention (Fig. 1B, D). Ferrostatin-1 (Fer-1) is a potent and selective inhibitor of ferroptosis, which significantly rescued the DOX-induced reductions in FS% and EF% in mice. Notably, WGX50 showed almost equivalent efficacy compared to Fer-1. Histological analysis showed that DOX-treated mice exhibited myocardial cell atrophy, enlarged intercellular gaps, and increased myocardial fibrosis compared to the control group. However, administration of Fer-1 or WGX50 mitigated these changes (Fig. 1E). Additionally, the levels of myocardial injury markers i.e. ANP, BNP, CK-MB, and cTnT in myocardial tissue homogenate of C57BL/6 mice in Fer-1 and WGX50 treated groups were also decreased significantly when compared to the control group (Fig. 1F, I). These results reveal that WGX50 can reduce DOX-induced myocardial insufficiency in mice, presumably involving ferroptosis. Our results further showed that WGX50 significantly reduced the increase of Fe^2+^ and MDA, and alleviated the decrease of GPX4 protein level in DOX-induced mouse myocardial tissue (Fig. 1J, L), showing the same effect as Fer-1. These findings suggest that WGX50 alleviates DOX-induced myocardial dysfunction through inhibiting ferroptosis.

### WGX50 alleviates DOX-induced mitochondrial damage and redox imbalance in C57BL/6 mice

**Fig. 2:**
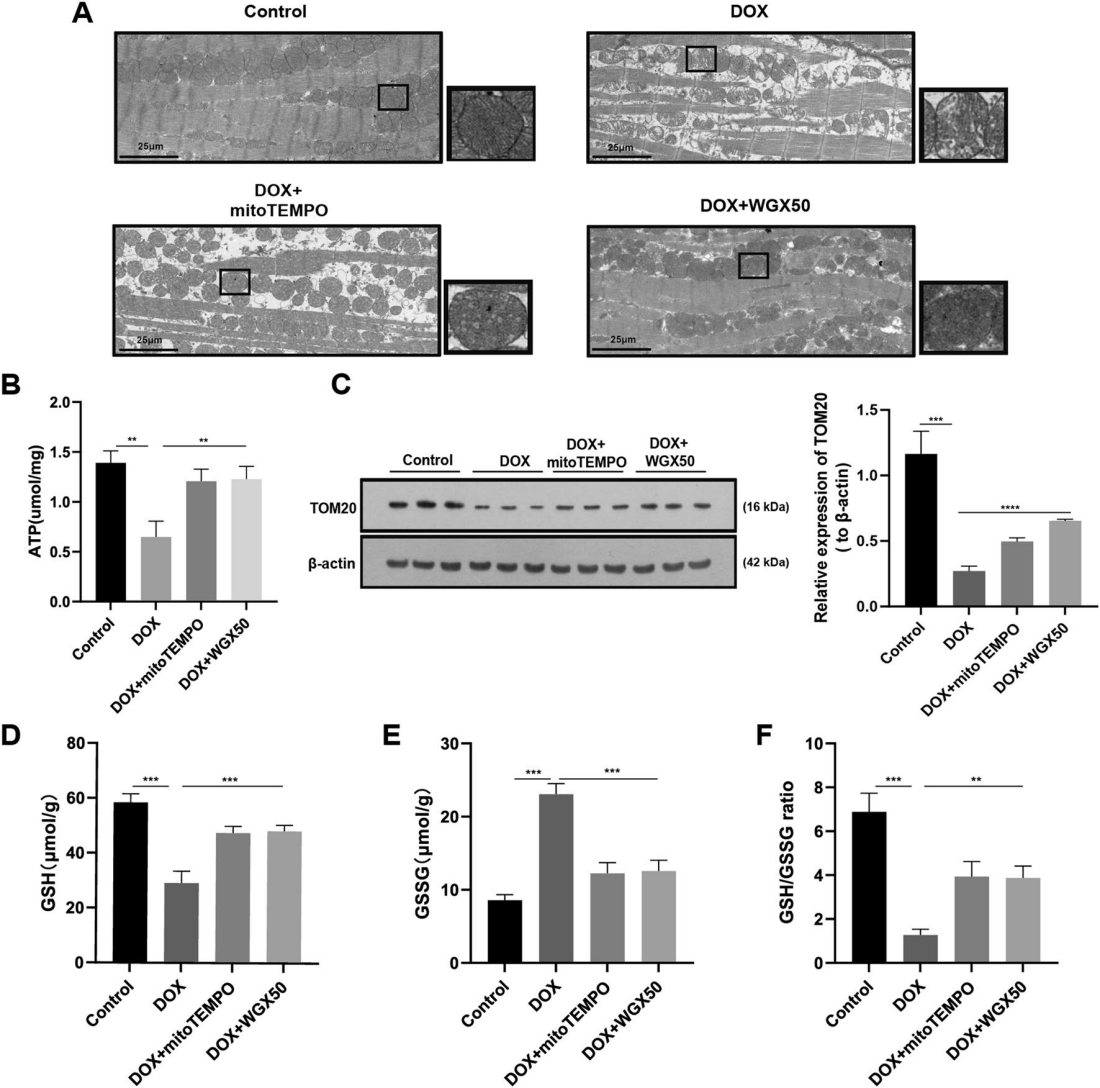
WGX50 alleviates DOX-induced mitochondrial damage and redox imbalance in C57BL/6 mice. **A** Transmission electron microscope images of mouse myocardium. Enlarged inserts at the right side of each image show typical morphology of mitochondria, “_” represents 25 µm. **B** The ATP level in mice myocardium. **C** Western blot bands show level of TOM20 protein in mouse hear tissues of different groups and the histogram is the relative expression of TOM20. Values are expressed as mean ± SD from three individual experiments (n = 3). **D**–**F** The levels of GSH, GSSG, and the GSH/GSSG ratio in mice myocardium. **, and *** respectively means* p* < 0.01, and *p* < 0.001

Considering the critical role of mitochondria in ferroptosis, we evaluated the effect of WGX50 on mitochondria in mouse myocardia. First, transmission electron microscopy showed that some mitochondria of mice myocardial tissue in DOX group exhibited vacuolation, disordered and loose arrangement of cristae, and incomplete membrane (Fig. 2A). MitoTEMPO, a mitochondria-targeting antioxidant, maintained effectively the integrity of mitochondrial structure and improved morphological changes caused by DOX. Strikingly, WGX50 also improved mitochondria disarrangement by increasing the integral membrane, minimizing vacuolation, and restoring cristae organization. (Fig. 2A). Next, the ATP production, and mitochondrial membrane protein TOM20 expression level in myocardium samples were all profoundly decreased after DOX treatment (Fig. 2B, C). Furthermore, alterations in redox homeostasis were investigated. As data shows, DOX resulted in GSH exhaustion in myocardium as indicated by reducting in GSH content and GSH/GSSG ratio and increasing GSSG level as well (Fig. 2F). However, concomitant administration of WGX50 or mitoTEMPO mitigated the mitochondrial damage and redox imbalance induced by DOX in C57BL/6 mice myocardium (Fig. 2B–F).

### DOX induces injury and ferroptosis in HL-1 cardiomyocytes

**Fig. 3:**
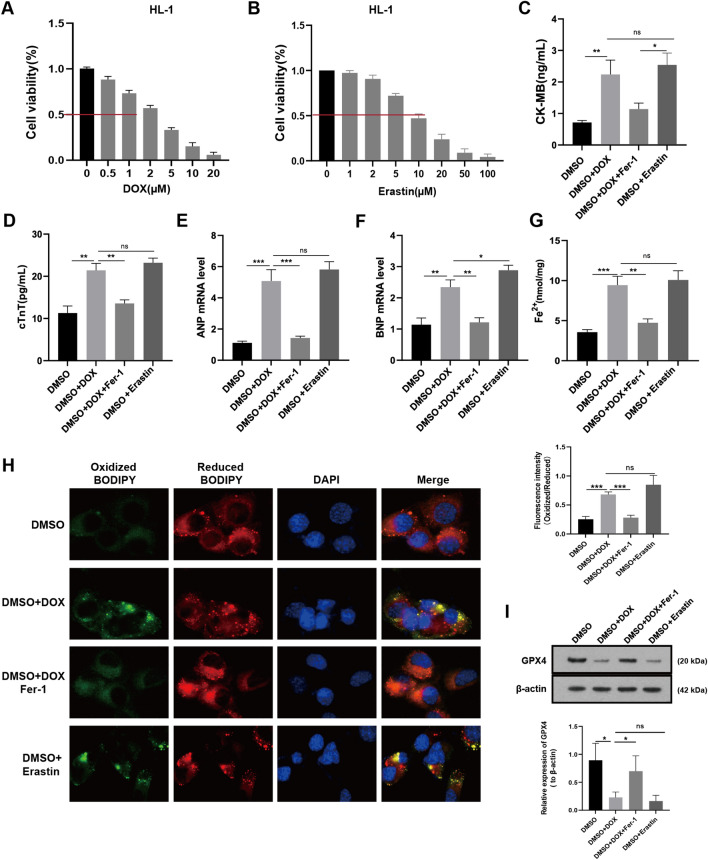
DOX-induced HL-1 cardiomyocytes injury and ferroptosis. **A** IC_50_ values of DOX in HL-1 cardiomyocytes. **B** IC_50_ values of Erastin in HL-1 cardiomyocytes. **C**–**G** The levels of CK-MB, cTnT, ANP, BNP, and Fe^2+^ in HL-1 cardiomyocytes. **H** C11-BODIPY 581/591 probe-stained images in HL-1 mouse cardiomyocytes, and the histogram of fluorescence intensity ratio (Oxidized/ Reduced) was calculated accordingly. **I** Western blot bands show protein level changes of GPX4 in HL-1 cardiomyocytes in different groups, the histogram represents relative quantification of GPX4. Values are expressed as mean ± SD from three individual experiments (n = 3). *, **, and *** respectively means *p* < 0.05, *p* < 0.01, and *p* < 0.001. “ns” means not statistically significant

To elucidate the underlying mechanisms of DOX-induced cardiomyocyte injury, we performed cellular experiments. We did IC50 experiments to determine the ideal concentrations of DOX and the ferroptosis inducer Erastin in HL-1 cardiomyocytes, which revealed values of 2 M and 10 M, respectively (Fig. 3A, B). Next, we measured the levels of CK-MB, cTnT, ANP, and BNP in HL-1 cell lysates. As indicated, the aforementioned indexes were all significantly elevated after DOX and Erastin intervention, whilst all those parameters were reversed after by Fer-1 treatment (Fig. 3C, F). These revealed that DOX intervention led to an increase in the levels of Fe^2+^ (Fig. 3G), which was similar to the changes associated with Erastin, and that were partially reversed by Fer-1. Staining images obtained using the C11-BODIPY 581/591 fluorescence probe showed increased green fluorescence in HL-1 cells after being treated with DOX and Erastin, indicating greater lipid ROS production in these cells. As expected, Fer-1 intervention effectively alleviated DOX-induced lipid ROS (Fig. 3H). Lastly, Fer-1 reversed the DOX-induced decrease in GPX4 expression (Fig. 3I). Taken together, these results suggest that DOX causes damage to cardiomyocytes by inducing ferroptosis.

### WGX50 inhibits DOX-induced damage by counteracting ferroptosis in HL-1 cells

**Fig. 4:**
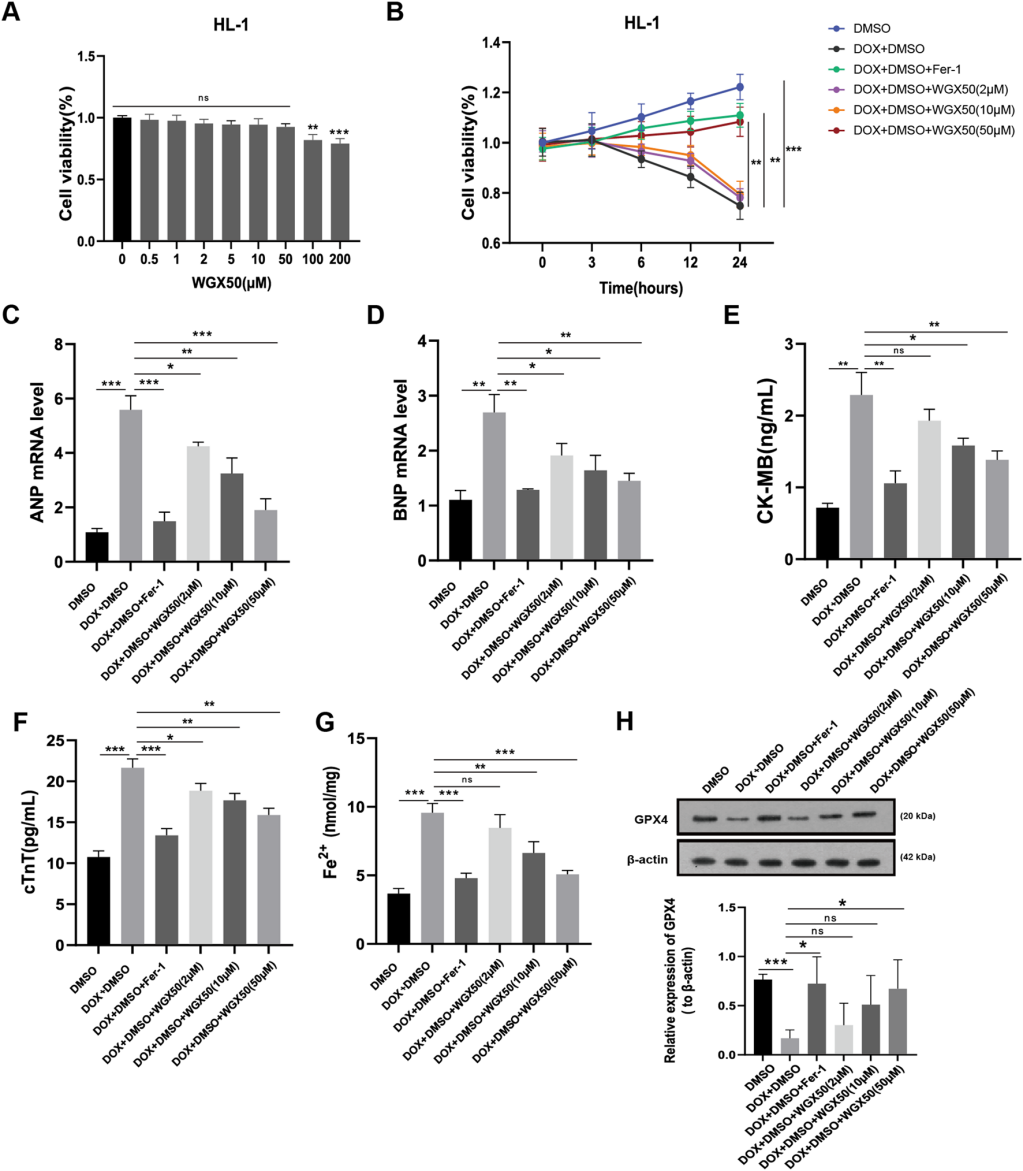
WGX50 alleviates DOX-induced cardiomyocytes injury by counteracting ferroptosis in HL-1 cells. **A** Cell viability assay of HL-1 cardiomyocytes when treated with 0, 0.5, 1, 2, 5, 10, 50, 100, 200 µM of WGX50 for 24 h. **B** HL-1 cells were cultured with 2 µM DOX in combination with or without 10 μM Fer-1 or with WGX50 at various concentrations (2, 10, 50 μM) for 3, 6, 12, and 24 h. Cell viability was measured by CCK8 method. **C**–**G** The levels of ANP, BNP, CK-MB, cTnT, and Fe^2+^ in HL-1 cardiomyocytes were determined by RT-qPCR and assay kits as available procedures across groups (**B**). **H** Western blot bands show relative expression level of GPX4 protein in HL-1 cardiomyocytes across groups as indicated in (**B**). The histogram illustrates the quantitative data of GPX4 protein after β-actin normalization. Values are expressed as mean ± SD from three individual experiments (n = 3). Values are expressed as mean ± SD from three individual experiments (n = 3). *, **, and *** respectively means *p* < 0.05, *p* < 0.01, and *p* < 0.001, “ns” means not statistically significant

We first evaluated the influence of WGX50 on HL-1 cell survival at various concentrations. Our results revealed no significant cytotoxicity at a concentration of 50 µM, whereas a decline in cell viability was observed at 100 µM (Fig. 4A). Therefore, we selected concentrations of 2 µM, 10 µM, and 50 µM of WGX50 to explore its potential protective effects in DOX-injured HL-1 cells. Notably, treatment with 50 µM of WGX50 showed a similarly prominent resistance to DOX-induced ferroptosis as Fer-1 did in HL-1 cardiomyocytes (Fig. 4B). Additionally, WGX50 remarkably inhibited the elevation of CK-MB, cTnT, ANP, BNP, and Fe^2+^ accumulation in HL-1 cells (Fig. 4C–G). More importantly, western blotting analysis revealed that WGX50 reversed DOX-induced reduction of GPX4 protein in cardiomyocytes at 50 µM concentration (Fig. 4H). Therefore, we speculate that WGX50 might be a promising drug that effectively alleviates DIC by inhibiting ferroptosis.

### DOX induces mitochondrial damage, mitochondrial ROS and lipid peroxidation in HL-1 cells

**Fig. 5:**
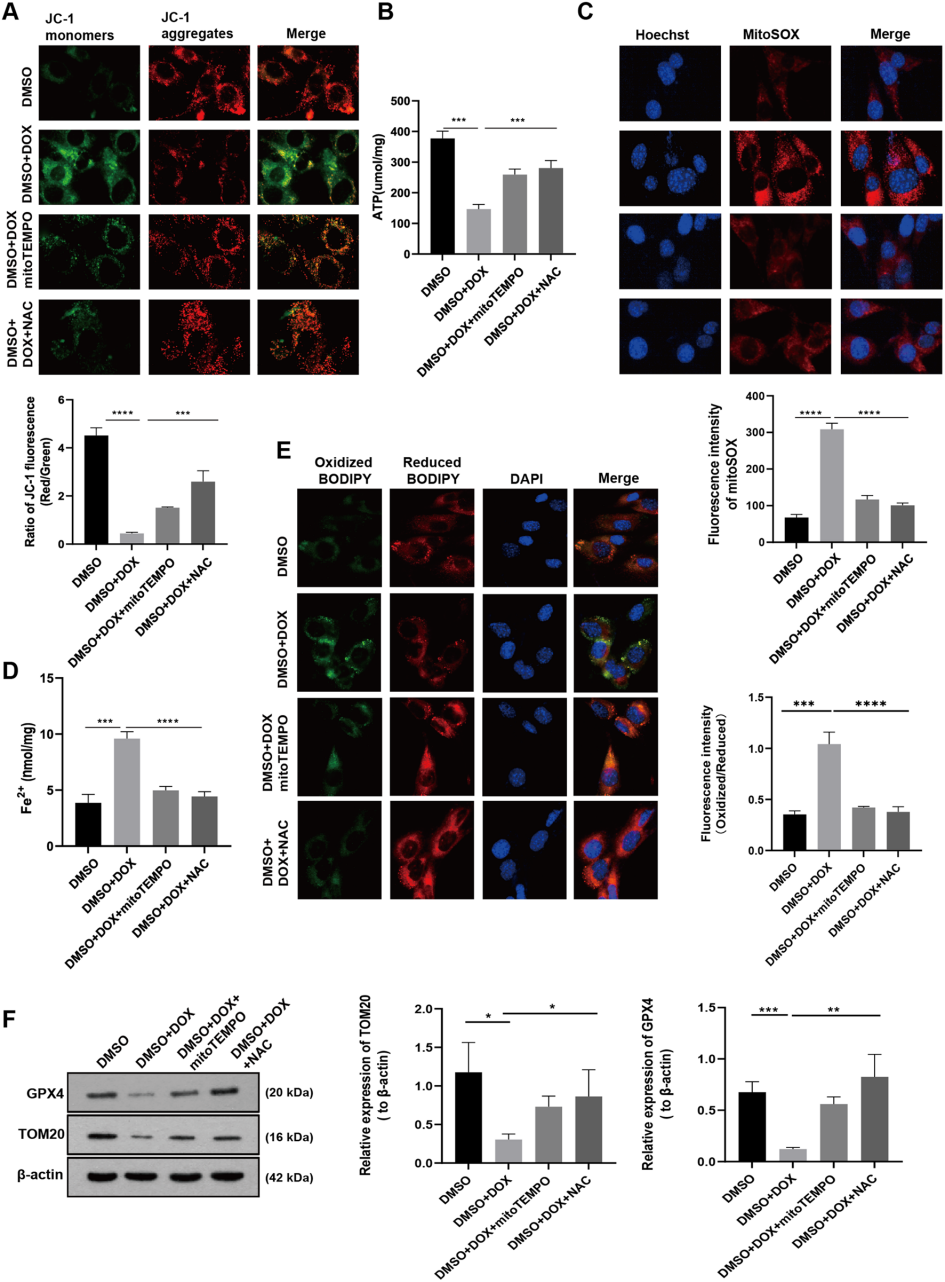
DOX induces mitochondrial damage, mitochondrial ROS and lipid peroxidation in HL-1 cells. **A** Mitochondrial membrane potential was measured using JC-1 fluorescent probe in HL-1 cardiomyocytes after 24-h treatment of DOX (2 μM) in combination with or without mitoTEMPO (10 μM) or NAC (5 μM). The histogram represents the ratio of JC-1 red to green fluorescence (Red/ Green). **B** ATP content in HL-1 mouse cardiomyocytes after treatments in (**A**). **C** Images of MitoSOX red dye was used to detect mitochondrial superoxide in HL-1 mouse cardiomyocytes across treatments as indicated in (**A**). The histogram shows MitoSOX fluorescence intensity. **D** The level of Fe^2+^ in HL-1 cardiomyocytes after being treated as method in (A) for 24 h. **E** Lipid peroxidation was measured using C11-BODIPY 581/591 probe in drug treated HL-1 cardiomyocytes as referred in (**A**). Histogram presents fluorescence intensity of oxidized BODIPY. **F** Western blot bands show expression of GPX4 and TOM20 protein in HL-1 cardiomyocytes in same conditions (**A**). Histograms show relative levels of GPX4 and TOM20 normalized by β-actin. Values are expressed as mean ± SD from three individual experiments (n = 3). *, **, and *** respectively means *p* < 0.05, *p* < 0.01, and *p* < 0.001

Subsequently, we further investigated the impact of DOX on mitochondria in HL-1 cells using various fluorescent tracing probes. Initially, we used the JC-1 fluorescent probe to detect mitochondrial membrane potential (MMP) changes in HL-1 cells. Our results show that the probes in the DOX treatment group were mainly in the form of green fluorescent monomers. In contrast, after the intervention of mitoTEMPO and antioxidant N-Acetyl-L-cysteine (NAC), they were converted into red fluorescent polymers, indicating the destruction of MMP in HL-1 cells by DOX (Fig. 5A). Figure 5B illustrates that DOX reduces mitochondrial ATP synthesis, which can be reversed by employing ROS scavengers. Red fluorescence intensity of MitoSOX, a mitochondria specific superoxide indicator, in DOX treatment group was significantly enhanced, whereas both mitoTEMPO and NAC reduced red fluorescence intensity in HL-1 cells (Fig. 5C). NAC is a potent intracellular ROS scavenger. These results indicate that DOX induced mitochondrial damage results in a large amount of ROS in mitochondria causing injury to cardiomyocytes. Moreover, the Fe^2+^ level was decreased in HL-1cells after DOX treatment for 24 h but effectively reversed by mitoTEMPO and NAC (Fig. 5D). Next, the C11-BODIPY 581/591 probe was used to detect the level of lipid ROS in HL-1 cells. It was found that DOX-treated cardiomyocytes exhibited a fluorescence shift from red to green, indicating a significant increase in lipid ROS levels in those cells. Conversely, the antioxidants MitoTEMPO and NAC maintained a low level of membrane oxidation in the cells (Fig. 5E). Finally, we found that antioxidants also partially remedied the DOX-induced reduction in GPX4 and mitochondrial membrane protein TOM20 (Fig. 5F). These findings indicate that protecting mitochondrial function and maintaining redox balance may be an efficient strategy for fighting DIC.

### WGX50 mitigates DOX-induced mitochondrial damage and lipid peroxidation in rodent cardiomyocytes

**Fig. 6:**
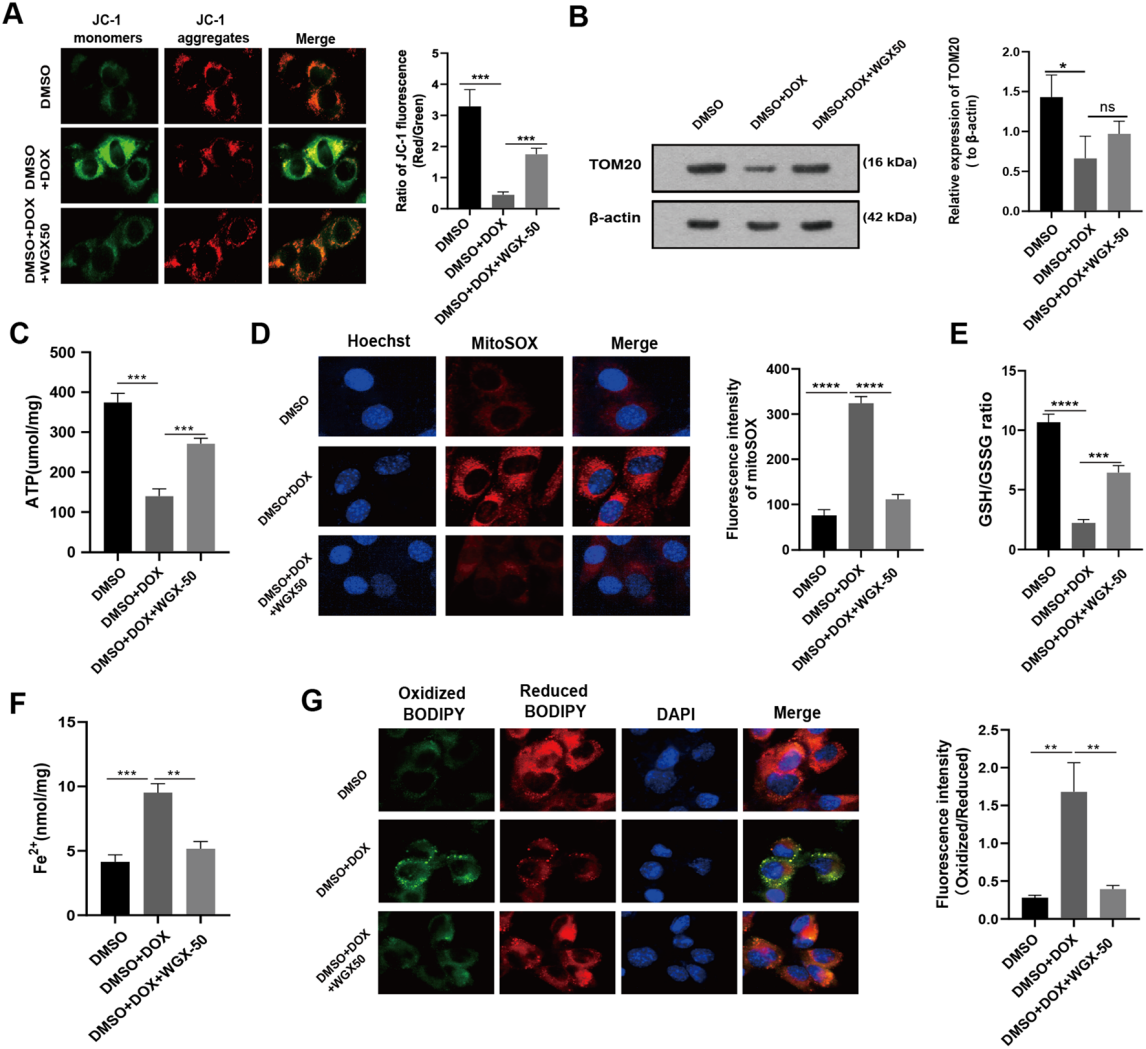
WGX50 mitigates DOX-induced mitochondrial damage and lipid peroxidation in HL-1 cells. **A** Mitochondrial membrane potential was measured using JC-1 fluorescent probe in the cultured HL-1 cells with 50 μM of WGX50 for 24 h, and the histogram represents the ratio of JC-1 red to green fluorescence (Red/ Green). **B** Western blot bands show expression level of TOM20 protein in HL-1 cells that were treated with DOX (2 μM) and WGX50 (50 μM) for 24 h. Histogram shows relative quantification of TOM20 with β-actin normalization. **C** The level of ATP in HL-1 cells. **D** Images of MitoSOX stained HL-1 cells, and the histogram shows MitoSOX fluorescence. **E** The GSH/GSSG ratio in HL-1 cells. **F** The levels of Fe^2+^ in the HL-1 cells. **G** Images of C11-BODIPY 581/591 stained HL-1 cells. Histogram presents fluorescence intensity of oxidized BODIPY (Oxidized/ Reduced). Values are expressed as mean ± SD from three independent experiments (n = 3). **, *** and **** respectively means *p* < 0.01, *p* < 0.001 and *p* < 0.0001

Afterward, we verified the protective effect of WGX50 on DOX-induced mitochondrial damage in HL-1 cardiomyocytes. WGX50 prevented cells from DOX-induced unbalance of MMP and decrease in ATP production, and TOM20 levels (Fig. 6A–C). WGX50 also reduced the red fluorescence triggered by DOX under mitoSOX staining (Fig. 6D) and increased the GSH/GSSG ratio (Fig. 6E), showing strong antioxidant capacity. Excitingly, WGX50 reduced Fe^2+^ accumulation (Fig. 6F) and green fluorescence intensity stimulated by DOX in C11-BODIPY 581/591 fluorescence staining, lowered lipid peroxidation and thus leading to less severe ferroptosis (Fig. 6G). Besides, a second set of the measurements in H9C2 cells was consistent with results obtained from HL-1 cells, which further supporting our hypothesis (Additional file 1: Fig. S2).

### WGX50 has a strong effect on reducing mitochondrial damage and inhibiting ferroptosis

**Fig. 7:**
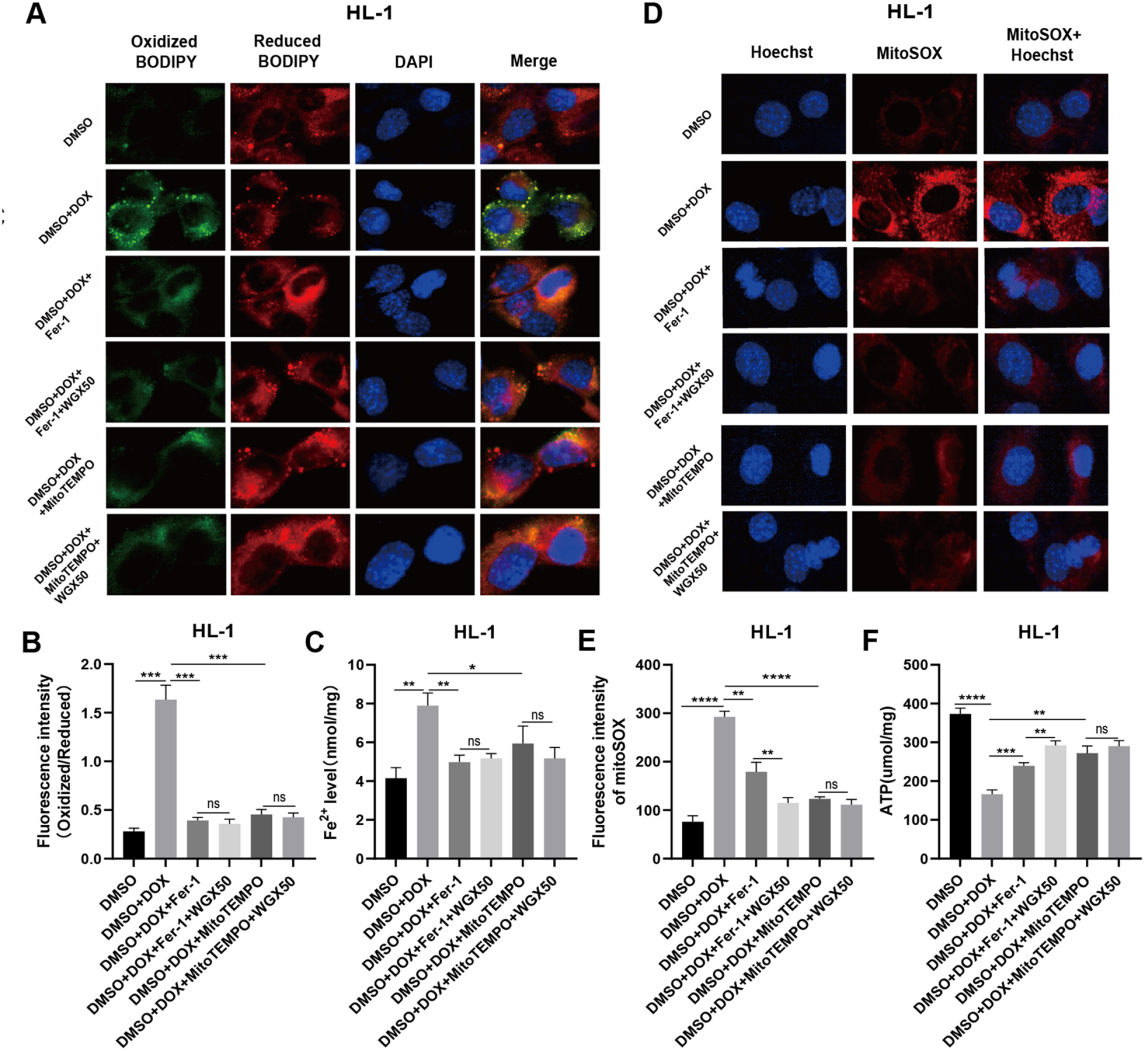
WGX50 has a strong effect on reducing mitochondrial damage and inhibiting ferroptosis. **A** Images of C11-BODIPY 581/591 stained HL-1 cells. **B** Histogram presents fluorescence intensity of oxidized BODIPY (Oxidized/ Reduced). **C** The levels of Fe^2+^ in the HL-1 cells. **D** Images of MitoSOX stained HL-1 cells, and the histogram **E** shows MitoSOX fluorescence intensity. **F** The level of ATP in HL-1 cells. Values are expressed as mean ± SD from three independent experiments (n = 3). *,**, *** and **** respectively means* p* < 0.05, *p* < 0.01, *p* < 0.001 and *p* < 0.0001, “ns” means no significance

We further evaluated the effect of WGX50 on DOX-induced ferroptosis and mitochondrial oxidative stress in the presence of the ferroptosis inhibitor Fer-1 and the mitochondrial antioxidant MitoTEMPO. We observed that WGX50 did not significantly diminish the DOX-induced rise in lipid ROS and iron levels in cardiomyocytes, meaning that WGX50 may have the same ferroptosis inhibitory effect as Fer-1 and MitoTEMPO (Fig. 7A–C). When we assessed mitochondrial ROS and ATP production, we noticed that WGX50 could considerably lessen DOX-induced increases in mitochondrial ROS and decreases in ATP levels in the cardiomyocyte on the basis of Fer-1 intervention, but not after MitoTEMPO intervention (Fig. 7D–F). This indicated that WGX50 had a stronger mitochondrial protective effect than Fer-1, but a comparable effect to MitoTEMPO.

### Molecular docking and simulation analysis of WGX50-GPX4 complex

**Fig. 8:**
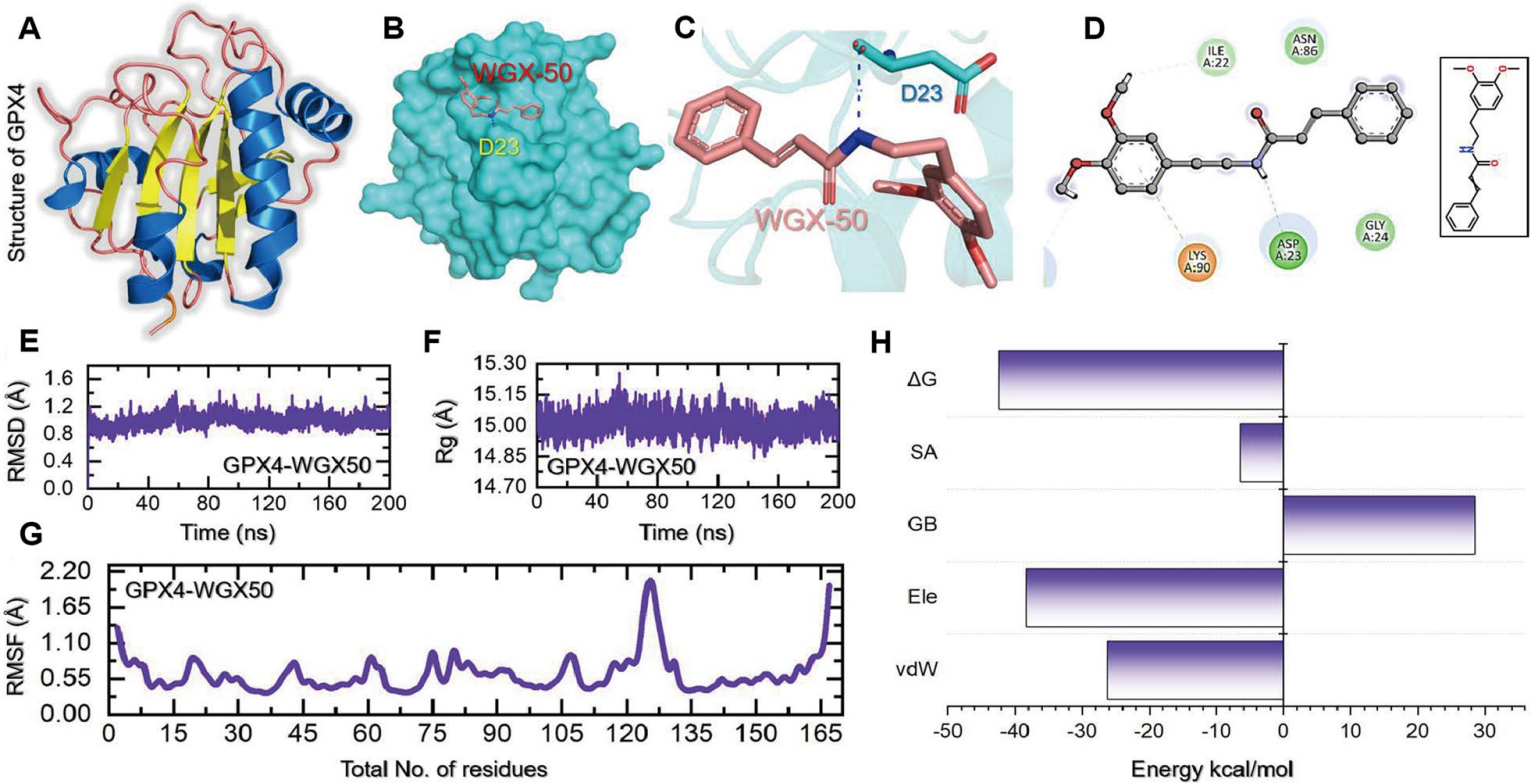
Molecular docking and simulation analysis of WGX50-GPX4 complex. **A**–**D** show the structure of GPX4, the binding of WGX50 as surface, sticks representation of the interaction between WGX50 and Asp23, and the 2D interaction pattern of WGX50-GPX4. **E**–**H** show the simulation analysis including RMSD (root mean square deviation), RMSF (root mean square fluctuation), Rg (radius of gyration) and binding free energy (BFE)

Among the hydrogen bonds were those at Ile22, Asp23, and Glu87, while the only pie-cation interaction was established by Lys90 amino acid of GPX40. The structure of GPX4, the binding of WGX50 as surface, sticks representation of the interaction between WGX50 and Asp23 while the 2D interaction pattern are shown in Fig. 8A–D. We further validated the binding stability through molecular simulation. The dynamic stability calculated as root mean square deviation (RMSD) of the WGX50-GPX4 demonstrated a very stable binding of WGX50 (Fig. 8E). An average RMSD was calculated to be 1.0 Å for the WGX50-GPX4. Similarly, the radius of gyration (Rg) also demonstrated that this complex i.e. WGX50-GPX4 possesses stable dynamics with no major deviations and thus maintains the stable binding behavior by demonstrating minimal unbinding events (Fig. 8F). Interestingly, the region Ile22, Asp23, Asn87 and Lys90 which comes in direct interaction with WGX50 determined minimal fluctuation when compared to the other regions. This shows that the binding of WGX50 stabilizes the internal fluctuation of these residues and therefore induces the functionality required for GPX4 activation. The RMSF (root mean square fluctuation) results are shown in Fig. 8G. To re-evaluate the binding conformation and docking predictions we also calculated the binding free energy (BFE) by using MM/GBSA approach. The BFE results revealed a vdW of − 38.27 kcal/mol, electrostatic energy of − 26.25 kcal/mol while the total binding free energy was estimated to be − 42.35 kcal/mol (Fig. 8H). This shows that WGX50 exhibits stronger binding potencies and therefore potentially plays a significant role in the activation of GPX4.

## Discussion

WGX50, a *Zanthoxylum bungeanum* Maxim extract that has been investigated for many years, has multiple activities such as anti-inflammatory, antioxidant, and neuroprotective actions. Our study has revealed WGX50’s capability of protecting myocardial injury from DOX inteversion both in vitro and in vivo. More specifically, WGX50 attenuated DOX-induced cardiac impairments in C57BL/6 mice, and recused DOX-induced ferrous irons, lipid peroxidation, excessive mitochondrial ROS (mtROS), glutathione (GSH) exhaustion, and loss of GPX4 protein in mouse and rat cardiomyocytes. WGX50 was demonstrated good antioxidant property in cardiomyocytes against DOX-induced mitochondrial oxidative stress, preserving mitochondrial structure, membrane integrity, and function while acting as an mtROS-mediated anti-ferroptosis agent in cells.

As tested, the safety of WGX50 was acceptable. There is no significant toxicity in major organs such as liver, kidney, lung, and spleen in mice after being injected intraperitoneally with 1 mg/kg of WGX50 daily for 4 weeks. However, WGX50 was reported to be readily metabolized in rat by liver and kidney and was excreted completely within 4 h post oral (PO) [34] and intravenous (IV, unpublished data) administration. Therefore, further safety assessments of WGX50 are essential for the other applications in case of increased dose, and all our results reported here might vary accordingly.

DOX induces cardiotoxicity that initially manifests as subclinical myocardial damage and leads to an early asymptomatic reduction in left ventricular ejection fraction (LVEF, EF%), and eventually to intractable heart failure [8]. Therefore, echocardiography was utilized for cardiac function quantification. Interestingly, WGX50 significantly recovered reductions by DOX in LVEF and left ventricular systolic fraction (LVFS, FS%) nearly to the basal normal level in mice, which clearly is indicative of its excellent action in cardiac function. These data motivated us to examine the tissue changes by using HE and Masson staining and to perform qRT-PCR for key myocardial injury markers i.e. ANP, BNP, CK-MB, and cTnT in myocardial tissue homogenates. Surprisingly, WGX50 consistently demonstrated similar outcomes to Fer-1 in terms of reducing cardiac tissue injury and fibrosis in mice. Ferrostatin-1 (Fer-1) is a potent and selective inhibitor of ferroptosis. Ferroptosis, as demonstrated by Kai Hou and Yilong Wang et al. [12, 13], is a form of cell death that plays a crucial role in DIC [15]. Meanwhile, ferroptosis can induce cardiac fibrosis [49], which may be the target of WGX50 to inhibit cardiac fibrosis in mice.

Anthracycline-induced ferroptosis is closely related to the blockade of iron metabolism. Previous studies suggested that high levels of iron enhance cardiotoxicity in DOX-treated mice, leading to body weight loss and increased mortality [50]. Of note, glutathione peroxidase 4 (GPX4) is a crucial marker of ferroptosis that functions as the end terminator catalyzing lethal lipid peroxide into nontoxic lipid alcohols [12, 39, 51]. We measured Fe^2+^ and MDA, a crucial product of lipid peroxidation, in myocardial tissue specimens. Conceivably, DOX treatment does lead to the expected terrible changes in these measurements. Importantly, WGX50 significantly reduced the increase of Fe^2+^ and MDA exhibiting the same effects as Fer-1 did. Moreover, WGX50 alleviated DOX-induced decrease in GPX4 at protein levels, which suggested WGX50 might also regulate the activity of GPX4. As reported, GPX4 activity was depressed upon DOX administration [12]. So we performed molecular docking of WGX50 with the active site of GPX4 protein and conducted an unbiased fully atomistic simulation in parallel for the WGX50-GPX4 to uncover the interaction mode of this complex. As results indicate, WGX50 activated GPX4 through a direct interaction with Asp23 which had been previously reported to be essential for GPX4 activation and enhanced functionality. Yuan et al. reported that PKUMDL-LC-102 activates GPX4 by interacting with Asp23 [39, 51]. In a cellular assay, when Asp23 was mutated with Ala23 the loss of activation activity was observed in that mutant, and a concentration of more than 150% was required for the activation [39]. Interestingly, our results corroborate with the previous findings, which demonstrates that WGX50 particularly targets Asp23 residue and thus allosterically activates GPX4. Subsequently, we will take more effort on GPX4 activity assay studies, particularly in three major isoforms, namely the mitochondrial, cytosolic, and nuclear GPX4.

Given the vital role of mitochondria in DIC, we examined the morphology and functioning of mitochondria in mice myocardium. Hopefully, WGX50 greatly improved mitochondria disarrangement with a more integral membrane, less vacuolation, and much more well-organized cristae as evidenced by transmission electron microscopy analysis. On the other hand, DOX-induced lower expression of the mitochondrial membrane protein TOM20 in mouse myocardium was dramatically increased by WGX50. When it comes to mitochondria function, we tested ATP production, as expected, WGX50 facilitated the generation of ATP whilst DOX lowered it significantly. Mitochondria-targeted antioxidant mitoTEMPO has been reported to alleviate DOX-induced cardiac injury [38], as expected, it maintained effectively the integrity of mitochondrial membrane structure and improved ATP producing power. Our data proved the almost equivalent potency of WGX50 as mitoTEMPO, but it still suggested a noticeable impairment in mitochondrial protein import. TOM20 mainly functions as an outer membrane translocase [52] that is responsible for importing preproteins into mitochondrial inner sides from cytoplasm. However, both WGX50 and mitoTEMPO did not increase TOM20 protein level to the normal basal line. Nevertheless, for upcoming studies in this context, we could use an AIF deleted mice model that recapitulates multiple defects in mitochondria such as respiratory chain defects, aberrant fragmentation, cristae disruption etcetera [53].

Tadokoro et al. reported previously that mitochondrial dependent iron death played an important role in DIC [16], and our data also demonstrated mtROS-mediated ferroptosis in DIC. There are few DOX-induced ferroptosis models in HL-1 cells. Ferroptosis was previously investigated by monitoring iron, MDA, ROS, GSH, and MMP [54], but it was induced by palmitic acid insult. Clearly, other vital hallmarks were not included there. In this paper, we found that DOX led to mitochondrial damage in cardiomyocytes, produced a large amount of mtROS, and induced lipid ROS, thereby triggering ferroptosis. Most importantly, WGX50 ameliorated DIC by inhibiting mtROS and lipid ROS and rescued the ferroptosis.

In HL-1 cells, as reported by Xiaowei Zhu et al. in 2022 [55], 1 μM of DOX was sufficient to down regulate SLC7A11, the catalytic unit of system XC-, which could be profound for cystine uptake, and therefore limiting the biogenesis of GSH. In this context, however, the role of WGX50 in XC- system remains unclear if simply reexamining alterations in GSH and GSSG as we presented in this work. Recently, a structure of erastin-bound xCT-4F2hc complex has been released (PDB: 7EPZ) [56], and we insist that with increasing improvements in computational biology, the specific mechanisms of WGX50 would be resolved soon in silico and then be validated in biological systems.

Dexrazoxane, the only accessible ameliorant in clinics, significantly prevents DIC by depleting mitochondrial iron in the myocardium [17]. However, Dexrazoxane has the drawback of depleting topoisomerase II, counteracting DOX's anticancer effect, and it also increases the risk of acute myeloid leukemia and myelodysplastic syndrome [57].

Nevertheless, WGX50 could be a potent alternative drug to Dexrazoxane. Overall, we reported here for the first time that WGX50 has mtROS dependent anti-ferroptosis effects in mice with doxorubicin induced cardiotoxicity. We therefore suspect that WGX50 regimen could help to alleviate cardiotoxicity while maximizing the antitumor effect of DOX.

Increasing evidence suggests that natural products and herbal extracts may be a promising intervention strategy for DIC. Daidzein [21], Salidrosid [22], Psoralidin [23], epigallocatechin-3-gallate [58], Ganoderma lucidum polysaccharides [59] have been demonstrated to be helpful in alleviating DOX-induced cardiac damage, but the specific targets of these molecules in reducing DIC still need to be further explored. Moreover, the components and pharmacological mechanisms of herbal medicines are complicated, which also need to be further determined.

While our study has introduced an innovative finding regarding the potential protective effects of WGX50 against DIC by inhibiting mitochondrial ROS and ferroptosis, it is important to acknowledge certain limitations. Firstly, although our research has demonstrated the ability of WGX50 to activate GPX4 expression and inhibit iron-dependent cell death in cardiomyocytes in response to DOX intervention at the cellular and animal levels, further exploration is required to elucidate the specific mechanisms through which WGX50 regulates GPX4, thereby identifying more precise targets. Secondly, extensive investigation is necessary to determine whether the co-administration of WGX50 with DOX adversely affects the latter's anti-tumor activity. In light of these considerations, we are committed to conducting further research into these aspects, aiming to gain a comprehensive understanding of the underlying mechanisms involved.

## Conclusion

According to our findings, WGX50 effectively preserves mitochondrial morphology and function, maintains cellular redox homeostasis, reduces mitochondrial ROS, and regulates lipid metabolism and ferroptosis, thereby protecting cardiomyocytes from DOX insult and alleviating DOX-induced cardiac injury. Therefore, we believe that WGX50 is a meaningful potential protective agent against cardiac damage.

### Supplementary Information

**Additional file 1:** **Figure S1.** In vivo toxicity assessment of WGX50 by histology. **A** The chemical structure formula of WGX50. **B** Histopathological analysis of major organs after treatment with 1 mg/kg of WGX50 for 28 days (n = 3). “_” represents 100 µm or 200 µm. **Figure S2.** WGX50 alleviates DOX-induced mitochondrial damage and lipid peroxidation in H9C2. **A** IC50 values of DOX in H9C2 cells. **B** Cell viability of H9C2 mouse cardiomyocytes treated with 0, 0.5, 1, 2, 5, 10, 50, 100, 200 µM of WGX50 for 24 h. **C**, **D** The level of CK-MB and cTNT in H9C2 cells were cultured with 1 µM DOX and 50 μM WGX50 for 24 h. **E** Mitochondrial membrane potential was measured using JC-1 fluorescent probe in the cultured H9C2 and the histogram of ratio of JC-1 fluorescence (Red/Green). **F** Western blot bands showing level of TOM20 protein in H9C2 cells were cultured with DOX and WGX50 for 24 h and the histogram of relative expression of TOM20. **G** The level of ATP in H9C2 cells. **H** Images of MitoSOX probe-stained H9C2 cells and the histogram of MitoSOX fluorescence. **I** The GSH/GSSG ratio in H9C2 cells. **J** The levels of Fe2 + in the H9C2 cells. **G** Images of C11-BODIPY 581/591 probe-stained H9C2 cells and the histogram of fluorescence intensity ratio (Oxidized/Reduced). Values are mean ± SD from three individual experiments. *, **, ***, and **** respectively means *p* < 0.05, *p* < 0.01, *p* < 0.001, and *p* < 0.0001, “ns” means no significance

## Data Availability

All data are available in the main text or the supplementary materials. Further inquiries can be addressed to the corresponding author.
