# Supplementary material for: WGX50 mitigates doxorubicin-induced cardiotoxicity through inhibition of mitochondrial ROS and ferroptosis
Source: J Transl Med. 2023 Nov 17;21:823. doi: 10.1186/s12967-023-04715-1 (PMC10655295; doi:10.1186/s12967-023-04715-1)
Supplement: Supplementary file 1 — Additional file 1: Figure S1. In vivo toxicity assessment of WGX50 by histology. A The chemical structure formula of WGX50. B Histopathological analysis of major organs after treatment with 1 mg/kg of WGX50 for 28 days (n = 3). “_” represents 100 µm or 200 µm. Figure S2. WGX50 alleviates DOX-induced mitochondrial damage and lipid peroxidation in H9C2. A IC50 values of DOX in H9C2 cells. B Cell viability of H9C2 mouse cardiomyocytes treated with 0, 0.5, 1, 2, 5, 10, 50, 100, 200 µM of WGX50 for 24 h. C, D The level of CK-MB and cTNT in H9C2 cells were cultured with 1 µM DOX and 50 μM WGX50 for 24 h. E Mitochondrial membrane potential was measured using JC-1 fluorescent probe in the cultured H9C2 and the histogram of ratio of JC-1 fluorescence (Red/Green). F Western blot bands showing level of TOM20 protein in H9C2 cells were cultured with DOX and WGX50 for 24 h and the histogram of relative expression of TOM20. G The level of ATP in H9C2 cells. H Images of MitoSOX probe-stained H9C2 cells and the histogram of MitoSOX fluorescence. I The GSH/GSSG ratio in H9C2 cells. J The levels of Fe2 + in the H9C2 cells. G Images of C11-BODIPY 581/591 probe-stained H9C2 cells and the histogram of fluorescence intensity ratio (Oxidized/Reduced). Values are mean ± SD from three individual experiments. *, **, ***, and **** respectively means p < 0.05, p < 0.01, p < 0.001, and p < 0.0001, “ns” means no significance [file 12967_2023_4715_MOESM1_ESM.docx]

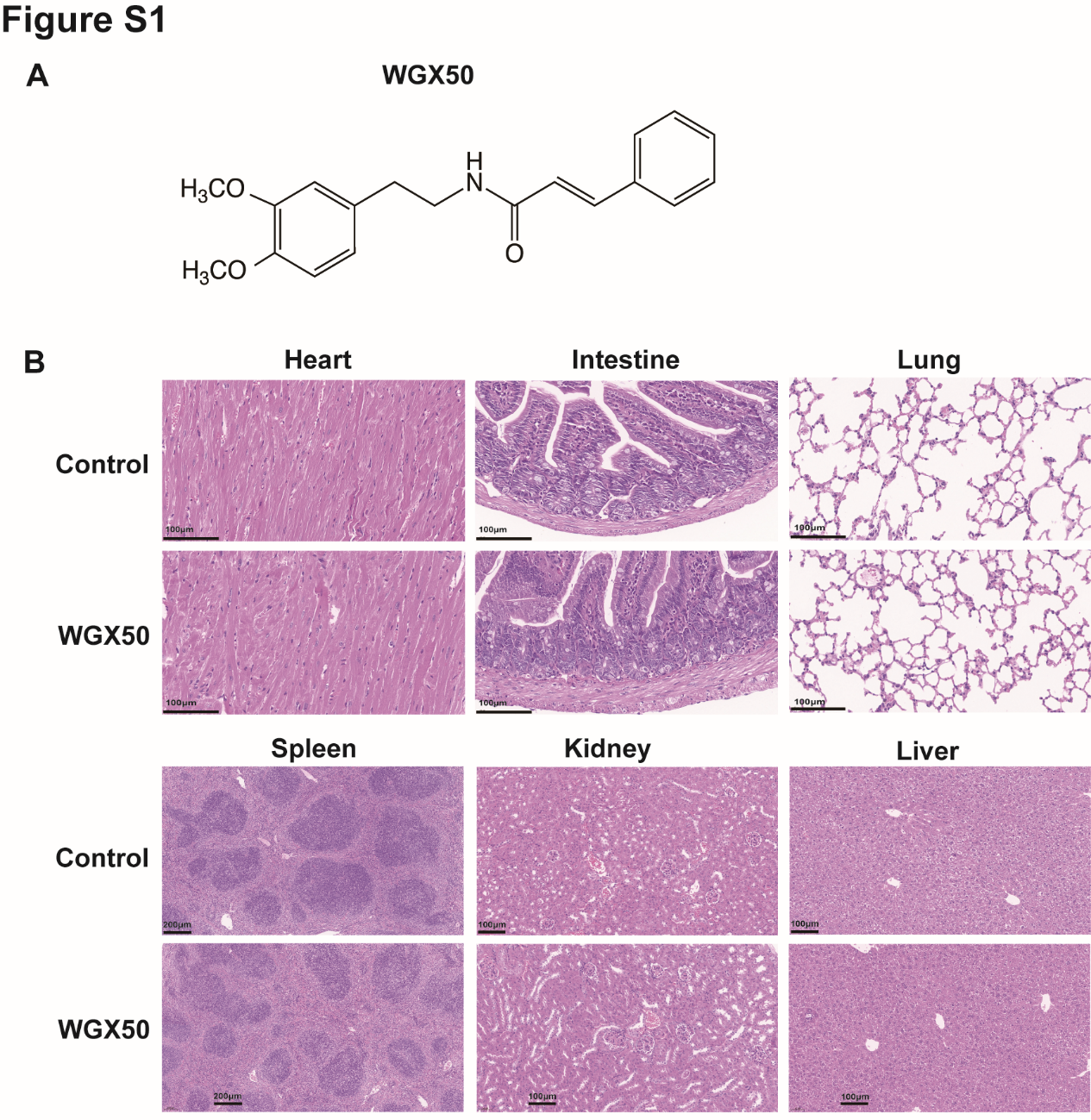


Figure S1. *In vivo* toxicity assessment of WGX50 by histology. (A) The chemical structure formula of WGX50. (B) Histopathological analysis of major organs after treatment with 1 mg/kg of WGX50 for 28 days (n = 3). “_” represents 100 µm or 200 µm.


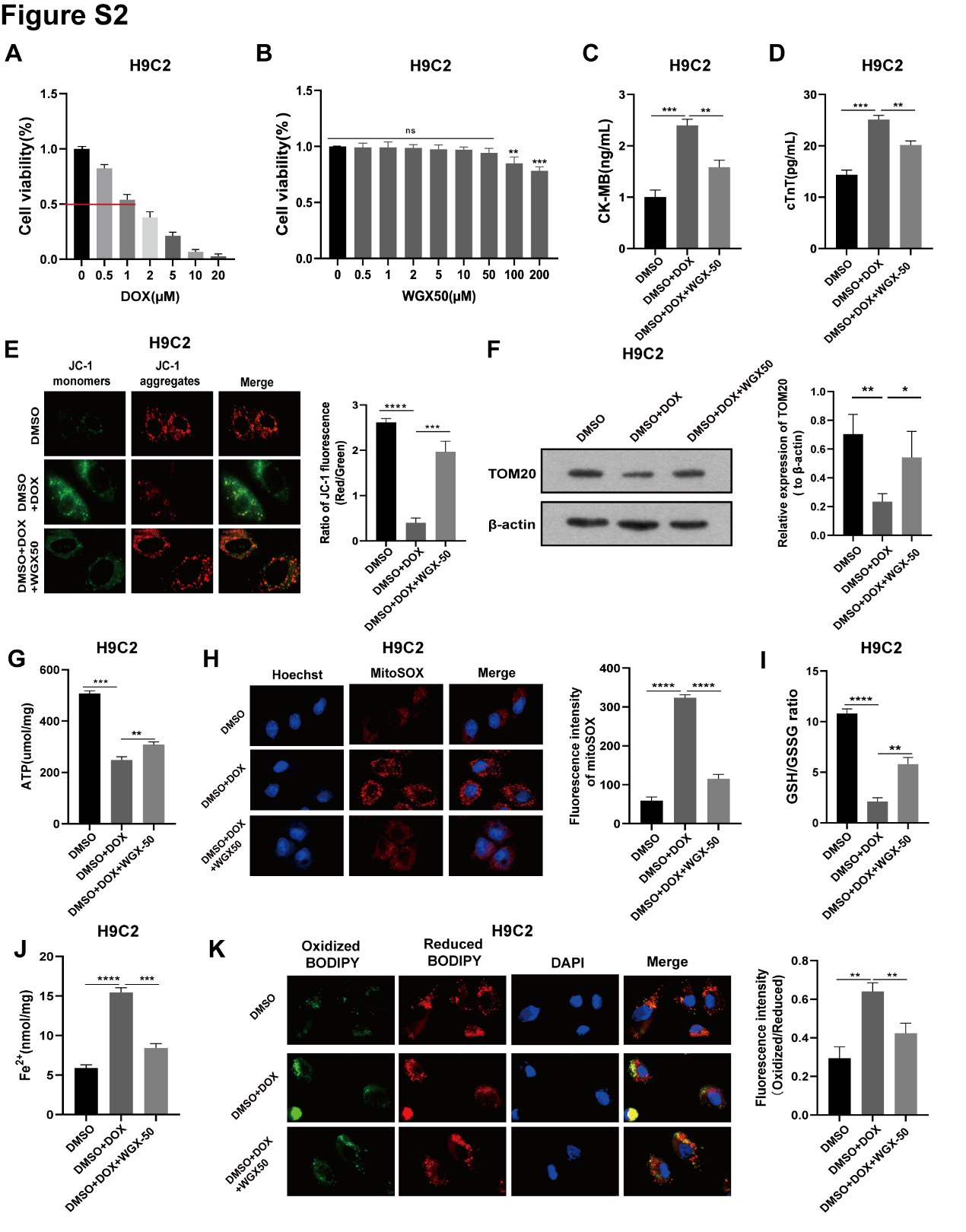


Figure S2. WGX50 alleviates DOX-induced mitochondrial damage and lipid peroxidation in HL-1. (A) IC50 values of DOX in H9C2 cells. (B) Cell viability of H9C2 mouse cardiomyocytes treated with 0, 0.5, 1, 2, 5, 10, 50, 100, 200µM of WGX50 for 24 h. (C-D) The level of CK-MB and cTNT in H9C2 cells were cultured with 1µM DOX and 50μM WGX50 for 24h. (E) Mitochondrial membrane potential was measured using JC-1 fluorescent probe in the cultured H9C2 and the histogram of ratio of JC-1 fluorescence (Red/Green). (F) Western blot bands showing level of TOM20 protein in H9C2 cells were cultured with DOX and WGX50 for 24h and the histogram of relative expression of TOM20. (G) The level of ATP in H9C2 cells. (H) Images of MitoSOX probe-stained H9C2 cells and the histogram of MitoSOX fluorescence. (I) The GSH/GSSG ratio in H9C2 cells. (J) The levels of Fe2+ in the H9C2 cells. (G) Images of C11-BODIPY 581/591 probe-stained H9C2 cells and the histogram of fluorescence intensity ratio (Oxidized/Reduced). Values are mean ± SD from three individual experiments. *，**, ***, and **** respectively means *p* < 0.05, *p* < 0.01, *p* < 0.001, and *p* < 0.0001, “ns” means no significance.
